# Supplementary material for: Are carbon emissions trading and green financial instruments synergistic? -Comprehensive quantitative research based on content analysis
Source: PLoS One. 2024 Mar 7;19(3):e0298601. doi: 10.1371/journal.pone.0298601 (PMC10919601; doi:10.1371/journal.pone.0298601)
Supplement: S1 Data — (ZIP) [file pone.0298601.s001.zip › Data/地级市Z_F工作报告.zip!/┴╔─■/│»╤⌠╩╨/└┤╫╘╣1⁄2╓┌║┼í╛┬φ┐╦╩2╛▌═°í┐_2007.docx]

各位代表：

　　我代表市人民政府向大会报告工作，请予审议，并请各位政协委员和其他列席人员提出意见。

过去一年工作回顾

　　2006年，我市经济建设和社会事业取得显著成就。

——经济发展既快又好。预计实现地区生产总值252.3亿元，增长17.6%，增幅列全省第三位；财政一般预算和国地税收入分别实现14.9亿元和33亿元，增长36.2%和15.9%，财政一般预算收入增幅列全省第一位；完成全社会固定资产投资139.7亿元，增长63.2%。全市经济发展呈现出速度较快、效益较好的态势。

　　——改革开放取得重大突破。全市长达5年之久的国有企业产权制度改革圆满结束，其他各项改革稳步推进。对外开放力度明显加大，引进域外资金80亿元，增长40%；实际利用外资2300万美元，增长近3倍，按可比口径超过“十五”时期的总和，增幅列全省第一位；出口创汇2.5亿美元，增长25%。

　　——人民生活水平进一步提高。完成城镇实名制就业6万人，城镇登记失业率控制在5.8%。预计城镇居民人均可支配收入和农民人均纯收入分别实现7425元和3380元，增长12.5%和12.6%；城乡居民储蓄存款余额达246.5亿元，增长12.6%。

　　——各项社会事业取得新进步。科技、教育、文化、卫生、体育等社会事业健康发展，实现了经济与社会的全面进步。

　　这些成就的取得，标志着市八届人大三次会议确定的各项目标任务已经圆满完成；标志着我市“十一五”旗开得胜、首战告捷；标志着朝阳综合实力又有新的提高，进一步增强了全市人民树立朝阳发展新形象的信心和勇气。

　　一年来，我们以科学发展观统领经济社会发展全局，主要做了以下几方面工作：

　　（一）积极贯彻宏观调控政策，产业结构调整取得新进展

　　我们积极适应宏观调控，进一步加大煤、电、油、运、资金和土地等生产要素的组织、协调和储备力度，加强经济运行调度，集中力量扶优培强一批骨干企业，迅速扭转了年初工业生产增长缓慢的局面，保持了全市工业经济较快增长。规模以上工业增加值实现72.3亿元，增长28%；新增销售收入亿元以上企业14户、5000万元以上企业20户、规模以上工业企业100户。

　　农业结构调整取得明显成效，农业三大主导产业增加值占第一产业增加值比重达到72.4%。全年新建大棚8.3万亩，创历史新高；畜牧业增加值实现8.16亿元，增长9.7%；完成造林绿化面积121.7万亩，启动建设京四高速公路朝阳段两翼绿化工程。大力实施农业产业化经营突破战略，新增规模以上龙头企业22户、省级龙头企业6户，新建各类农业园区62个，农产品加工产值实现40.4亿元、增长26.4%。

　　制定实施《朝阳市大力发展服务业推进服务业现代化行动纲要》，餐饮、商贸等传统服务业发展步伐加快，金融、保险、信息、邮政、通信等现代服务业进一步壮大。新完成房地产开发面积256.7万平方米，增长92%。预计社会消费品零售总额实现73.8亿元，增长15.2%。

　　（二）进一步拓展思路，招商引资成果创历史新高

　　我们坚持产业链招商、资源招商和其他招商方式一起抓，认真梳理产业链条，论证包装重点项目，以线结点、连线成面的招商引资新格局逐步形成。长三角、珠三角、大连、日本、韩国等国内外招商活动取得丰硕成果。我市应邀正式加入辽宁沿海城市经济联合体，区位认知的调整，使朝阳对外招商引资形象发生了根本性变化，为进一步加快发展赢得了新机遇。全年新引进和增资的超千万元以上项目170个，增长24%。

　　经过激烈竞争，成功引进中建通讯及高科技产品工业园，这是朝阳迄今为止最大的招商引资项目；豪德光彩贸易广场、百盛锆业、金达华神钛业、海西矿业、飞马汽车零部件、新元纺织、黑猫炭黑、鑫枫羊业等一批投资额度大、牵动作用强、对朝阳经济发展有重大支撑作用的大项目，全部启动建设；在省委、省政府的大力支持下，鞍山钢铁集团将与凌钢集团共同出资建设凌钢朝阳新区项目；燕山湖发电厂、大型煤化工等一批重点项目，前期准备工作进展顺利。

　　进一步加快各类园区建设。市开发区全年引进2000万元以上项目17个，实现域外招商引资5.5亿元，分别增长45.4%和37.5%；各县（市）区工业园区共新引进企业48户，总投资11亿元，基础设施投资2.85亿元。

（三）强力发展县域经济，新农村建设扎实推进

　　我们把县域经济作为新农村建设的重要载体，着力培育产业支撑，全面落实扶持政策，推动县域经济实现了快速发展。县域预计完成地区生产总值195亿元，增长27%，高于全市平均增幅9.4个百分点；完成地方财政一般预算收入8.7亿元，增长36.7%，五县（市）财政收入均超亿元。

　　率先在全省启动新农村建设工作。加大对农村公共事业的投入，农村固定资产投资增长2倍多。全市农村实现户户通电，57%的行政村通油路，村镇自来水普及率达21%，完成水土保持综合治理面积49.8万亩。7个市级重点镇和126个生态文明示范村的新农村建设规划编制完成，村容镇貌整治活动初见成效，农村生产生活环境明显改善。

（四）加大各项改革力度，加快体制机制创新步伐

　　对239户国有企业实施了产权制度改革，朝重资产重组和凌钢股份有限公司股权分置顺利完成；充分利用各种政策，化解了部分企业金融债务，改制企业大多焕发了生机活力。农村土地二轮延包工作基本完成，动物卫生管理体制改革进展顺利，集体林产权制度改革全面启动。进一步深化行政审批制度改革，市本级行政许可项目集中办理率达到91%；全面清理行政事业性收费，减轻企业和群众负担8924万元。事业单位人员聘用工作全面完成，卫生、财政、投资、金融、国有资产管理等项改革不断深化。

　　积极推进全民创业，加快所有制结构调整，民营经济营业收入、增加值、上缴税金分别增长34.9%、50%和26.2%。

　　（五）大力推进城镇化进程，进一步改善城乡面貌

　　坚持规划先行，编制完成了城市近期建设规划、凤凰组团修建性详规、西部工业园协调规划；采取国际招标形式，拉开了城市总体规划新一轮修编帷幕。

　　我市获国家“水环境治理范例城市”称号，大凌河城区段综合整治工程获“中国人居环境范例奖”，二期工程进展顺利；阎王鼻子水库二期移民工作实现突破性进展；京四高速公路朝阳段全线开工，珠江路和二其营子公铁立交桥建成通车，城区出口路改造全面完成；新建、改造和维修城市道路83.6万平方米；新增城区绿地85万平方米；双塔古街、北塔博物馆、盘龙购物广场等工程全部竣工，燕都大厦、兴隆商厦、长途客运中心、体育馆、污水处理厂等项目全面开工。四座县城共新增建成区面积11.2平方公里，朝阳县柳城园区建设取得实质性进展；重点小城镇建设步伐加快，辐射作用进一步增强。全市城镇化率提高2个百分点。

　　强力推进环境综合整治百日行动，市容市貌得到改善。主要污染物排放量继续下降，城乡空气质量又有新提高。

　　创建中国优秀旅游城市工作顺利通过国家验收。

　　（六）全心全意为民办实事，提高人民群众生活水平和质量

　　关心群众冷暖、解决涉及人民群众切身利益的问题，是各级政府义不容辞的责任。市政府在八届人大三次会议上承诺的十件实事已经全部落实：一是市区北大街、锦承铁路两侧和北票采煤沉陷区棚户区改造工程全部完成，新建安置楼65.6万平方米，8648户棚户区居民喜迁新居。二是落实整村推进项目村200个，是历史上最多的一年；规划新建移民小区38个，搬迁移民1014户；深入推进户有稳定收入项目工作，14万贫困人口实现稳定温饱。三是全面推行新型农村合作医疗，以村、以户为单位参合率分别达到100%和84%。四是多渠道开发就业岗位，确保了有就业能力和就业愿望的零就业家庭在20天内至少一人稳定就业。五是出资7076.5万元，对义务教育阶段所有困难家庭学生实施了“两免一补”，受益学生41万人次。六是动员各方面力量为7680户无电视农户捐赠电视机，20户以上自然村屯全部通广播电视。七是城乡大病救助制度运行良好，23.25万人口得到医疗救助。八是完成“城中村”改造10万平方米。九是新建九年一贯制寄宿学校16所，调整中小学101所（处），改造农村中小学D级险房8.6万平方米。十是企业职工月最低工资标准由350元上调到420元；提高了低保、养老保险保障标准和机关事业单位工作人员工资，受益人口达到37.6万人。

　　继续强化社保体系建设，养老、医疗、失业、工伤和生育保险覆盖面进一步扩大。建设农村饮用水工程120处，解决了4万人饮水困难问题。筹资700多万元，扶助2240名贫困大学生顺利入学。实施农村贫困户安居工程，完成4952户农村贫困户危房修建任务。多方筹措资金，提高了城镇困难群众取暖补贴标准。

　　（七）注重统筹兼顾，加快社会事业发展

　　制定实施《朝阳市中长期科技发展规划纲要》，成功举办首届产学研合作经验交流暨农产品、矿产资源深加工洽谈会，全年高新技术产品增加值预计完成11.5亿元，增长76%。

　　突出抓好义务教育特别是农村义务教育，“普九”成果进一步巩固和提高；新增城镇普通高中校舍面积15.1万平方米；全面实施县级职教中心内涵质量提升工程，职业教育实际招生增长10.4%。

　　深入开展“凌河之夏”等群众文化活动，启动实施“乡有文化站、村有活动室、组有文化活动中心户”工程；成功承办全国亿万老年人健身活动大会，我市健儿在省十运会上取得金牌总数第七、重竞技团体总分第一的好成绩，全民健身活动日益普及，文化体育事业活跃繁荣。

　　制定实施《朝阳市突发公共卫生事件应急预案》和《朝阳市突发公共卫生事件医疗救援应急行动方案》，公共卫生应急体系逐步完善；疾病防控有效加强，药品市场管理日趋规范。

　　人口和计划生育工作不断加强，各项控制指标全部完成省下达计划。民族地区经济社会实现新发展，民族宗教政策得到较好落实。农业普查工作顺利推进。审计、质量技术监督、档案、残疾人保障、气象、防震减灾抢险、地方志、外事侨务、老龄、妇女儿童、人防、民兵预备役等项事业也都取得了新成果。

　　（八）深入开展精神文明创建活动，进一步加强民主法制建设

　　围绕打造“信心朝阳”、树立朝阳发展新形象等主题，深入开展各类精神文明创建活动，干部群众的发展信心明显增强；认真贯彻《全民道德建设实施纲要》，全民思想道德素质不断提高。扎实推进双拥共建工作，军政军民关系更加和谐紧密。

　　自觉接受人大法律监督和工作监督、政协民主监督和人民群众的社会监督，建立了重大事项向人大常委会报告、向政协通报制度；加强同各民主党派、无党派人士和各人民团体的联系，认真听取各界人士的建议意见，民主渠道更为通畅；坚持重大事项决策前以不同形式广泛征求市民意见，政府决策的透明度进一步提高。全年办理人大代表建议67件，政协提案146件，办复率、满意和基本满意率均达到100%，解决率为近几年最高的一年。　 实施《朝阳市全面推进依法行政规划》，行政行为进一步规范；大力推行政务公开，政府采购、工程招投标全面实行阳光操作；强化行政效能监察，广泛开展政（行）风评议活动，机关效率明显提高；建立民心网，加强市民投诉中心建设，构筑了听取民意、反映民声的平台。坚持从源头上预防和治理腐败，廉政建设取得新成效。

　　坚持打防结合，严厉打击各种严重刑事犯罪和经济犯罪活动，不断加强社会治安综合治理，平安朝阳建设取得明显实效。认真执行《信访条例》，畅通信访渠道，信访秩序进一步规范。继续整顿市场经济秩序和矿业秩序，安全生产形势总体平稳。

　　各位代表！过去的一年，我们经受住了宏观环境趋紧、长期积累的结构性矛盾集中显现的严峻考验，全市经济保持了又快又好发展；我们战胜了严重干旱等自然灾害，农牧业生产实现了稳定增长；我们突破了区位条件不利、产业基础薄弱、资金瓶颈制约等因素的影响，招商引资工作上了一个新台阶；我们克服了市场价格波动、重点企业销售增长乏力等困难，工业经济实现了快速发展；我们下大力量解决就业形势紧、困难群体大、社会矛盾突出等诸多问题，就业再就业和对困难群体的帮扶援助工作成效显著，使朝阳经济和社会事业呈现蓬勃发展的新局面，树立了朝阳对外开放的良好形象。全市上下加快发展的信心更足，加快发展的氛围更浓，建设家乡的热情更高。虽然遇到的困难比预想的多，但取得的成绩比预期的好。在工作中，我们努力做到“四个坚持”：在目标定位上，坚持立加快发展之志，兴创新实干之风。自觉提高标杆，层层传递压力，在现实的困难面前，在发展的紧要关头，始终保持奋发有为的精神状态，做到了在应对挑战中把握主动，在扬长补短中增创优势，在超越自我中寻求突破，做到了速度能快则快、目标能超则超，实现了“十一五”的良好开局。在发展措施上，坚持以重点突破之策，取推动全局之功。我们把招商引资作为政府的第一位工作，把项目建设作为最主要的牵动手段，整合和挖掘矿产资源、企业资源、文化资源等优势，高频率、大密度地开展招商引资，促进了一批大项目落户朝阳；坚持把发展作为改革的第一取向，以算大账、算长远账、算全局账的眼光，积极引进战略投资者，致力于把企业做大做强；坚持高起点、高标准、大手笔搞好城市建设，拉开了城市框架，提升了城市品位。在工作落脚点上，坚持尽执政为民之责，办富民利民之事。我们在指导思想上明确：发展是硬道理、富民也是硬道理，发展要加快、富民也要加速。采取一系列措施，增加收入，扩大就业，纾解民困，最大程度地凝聚民心，让人民群众更快更多地享受到改革发展成果。在发展氛围上，坚持聚全市人民之力，成团结奋进之势。我们坚持重大事项向市委请示、向人大报告、与政协协商制度，重大决策注意倾听群众呼声，注意集中民智、发挥民力，形成了共同推进朝阳发展的强大合力。过去一年的实践证明，朝阳不愧是一个发展潜力巨大的地区，只要全市上下一心，敢想善为，扎实工作，就一定能够在辽西北高高扬起朝阳的旗帜，就一定能够谱写出新的篇章！

　　过去一年取得的成绩，是省委、省政府和市委正确领导的结果，是市人大、市政协监督支持的结果，是全市各族人民辛勤劳动、团结奋斗的结果。在此，我代表市人民政府，向全市广大工人、农民、知识分子、干部和离退休老同志，向各民主党派、各人民团体和社会各界人士，向驻朝解放军指战员和武警官兵，向中央和省驻朝单位，向所有关心和支持朝阳发展的朋友们，表示衷心的感谢并致以崇高的敬意！

　　在肯定成绩的同时，我们也清醒地看到，我市经济社会发展还存在许多困难和问题。主要是：经济结构不合理，工业过分或简单依赖几家大型骨干企业和矿产资源的现象还很突出，农业产业化和第三产业存在着总量小、增速慢、层次低等问题，在加快发展中推进产业结构优化升级任务十分艰巨；工业化推进速度还较慢，真正能够拉动经济发展的立市立县企业较少，企业自主创新能力不强，产业集群化发展不够；受宏观经济环境的影响，各种生产要素制约的矛盾逐步凸显，为推进招商引资和项目建设增加了难度；城镇化步伐不快，特别是基础设施不够健全，产业发展空间不足，还不能很好发挥以城带乡的功能；部分领域改革滞后，经济社会发展还存在许多体制性、机制性障碍，深化改革的任务比较繁重；经济社会发展“一条腿长、一条腿短”的问题还很突出，生态脆弱的问题尚未得到根本改善，协调发展的任务还很艰巨；农民持续增收、城镇居民就业持续增加的难度大，社会弱势群体生活还较困难，与构建和谐社会的要求还有较大差距。政府职能转变还不能很好地适应市场经济发展的要求，依法行政能力需进一步提高，有些部门和工作人员作风不扎实、工作效率低，等等。对此，我们一定高度重视，大力加以解决。

2007年政府工作安排

　　各位代表！新的一年里，我们要按照市第八次党代会和市委八届三次全会暨全市经济工作会议要求，坚持以科学发展观统领经济社会发展全局，深入实施“一主四化”核心战略，以深化改革、扩大开放为动力，强力推进产业结构升级，强力推进县域经济发展，强力推进和谐社会建设，高高扬起朝阳的旗帜，努力实现国民经济又好又快发展，加快树立朝阳发展新形象的进程。主要预期目标是：地区生产总值增长18%以上，力争突破300亿元；财政一般预算收入增长20%；固定资产投资增长50%以上，突破200亿元；城镇居民人均可支配收入和农民人均纯收入分别增长12%，城镇登记失业率控制在5%以内，各项社会事业与经济建设协调发展、同步提高。

　　为实现上述目标，重点做好以下六项工作：

（一）坚定不移地高强度开展招商引资工作，实现改革开放新突破

为了更好地振兴朝阳老工业基地，为了更多地吸引产业转移，为了更快地做大经济总量，必须强力推进招商引资。坚持产业链招商、资源招商和其他招商形式并用，按照全员招商、专业招商、以商招商、委托代理招商相结合的思路，下大力气搞好招商活动，采取机关干部挂职等办法，搞活招商方式，在优势产业链条上实现招商引资新突破，确保年内域外引资增长30%以上，实际利用外资增长25%。

　　支持创汇骨干企业建立国际营销网络，积极开拓汽车零部件和电子通讯等产品出口渠道，确保全市外贸出口增长25%。

　　加大招商引资载体建设，优化生产力布局。加快工业生产要素向园区聚集，积极引导和支持企业进园区发展，特别要鼓励支持城镇工业企业向园区实施扩张性、提高性搬迁改造，提高园区的投资强度和规模效益。继续按照“举债兴业、设施先行”的思路，扎实推进园区水电路等基础设施建设。

　　认真落实全省关于加快县域工业园区发展的各项政策，支持各类园区形成更大规模的扩张和有特色的产业集群，推动县域工业向规模化、集约化发展。

　　市开发区确保新增开发面积2平方公里，招商引资和固定资产投入均完成10亿元以上；省级高新技术园区、龙城现代农业园区年内正式挂牌运作，并在招商引资上取得新突破。

　　坚持以改革促开放，增强经济发展活力。按照深化产权多元化改革的要求，加快凌钢集团股份制改造步伐，完成浪马等企业国有法人股减持；采取行政推动与市场运作相结合的办法，加快推进企业联合重组步伐；紧紧抓住股市大发展的有利契机，运用资本手段引导和推动有条件的企业实现自主上市、借力上市。

　　大力开展全民创业，进一步优化民营经济发展环境，搭建银企沟通平台，认真落实各项扶持政策，确保民营经济营业收入和增加值分别增长30%以上。

　　巩固农村税费改革成果，积极稳妥地推进土地使用权流转，促进土地规模经营。全面完成集体林产权制度改革、动物卫生管理体制改革和国有农场体制改革。每县（市）选择2个乡（镇）、每区选择1个乡（镇）开展农村综合改革试点工作。

　　进一步完善公共财政制度，推进国库集中收付改革，加强财政监管和政府债务管理，提高财政资金使用效益。认真做好统一内外资企业所得税工作，强化税收征管，严格依率计征、依法纳税。继续深化事业单位人事制度改革，在分配制度改革和职称改革等方面实现新突破。

　　（二）进一步促进产业优化升级，推动增长方式转变

搞好产业基地建设，提高工业经济运行质量和效益。工业是我市经济发展的重中之重。强化工业经济运行调度，协调好资金、电力、土地、运力等生产要素，建立“旗林”企业市长直通车制度，及时帮助企业解决实际问题；通过整合各种发展资金、协调金融部门贷款、实施财政贴息等措施，重点扶持50个具有示范牵动作用的调整改造项目和50个高新技术产业项目，努力培植一批新的立市立县企业。全年新增超亿元企业12户、各类规模以上企业100户，工业增加值增长21%以上。

　　搞好产业基地建设，是今年全市加快产业结构优化升级步伐的重中之重。在精品钢材基地建设上，加快凌钢新区建设步伐，基本完成各项规划设计、主体工程土建和设备采购任务；搞好凌钢老厂3＃高炉改造项目；整合全市冶金、铸造企业资源，引导其产品升级换代。

　　在新型能源基地建设上，全力推进燕山湖发电厂、大型煤化工、甜高粱乙醇等项目，积极创造条件争取项目尽早开工上马；做好太阳能、风能开发利用的资源评估和管理，推进北票北塔风力发电厂和油页岩、煤层气资源开发等项目。

　　在农产品加工基地建设上，深入实施农业产业化经营突破战略，用好用活农业产业化建设资金，围绕大型产业化龙头企业开展“助推”行动，扶持壮大宏达牛业、鑫枫羊业等一批现有龙头企业，积极培育和引进一批新的龙头企业。

　　在汽车及汽车零部件基地建设上，抓好朝柴与美国万国集团合作的3升机改造、浪马100万套扩产改造、飞马车桥汽车零部件等一批重点项目，推动东风思益、一汽凌汽生产线尽快达产达效。

　　在矿产资源深加工基地建设上，重点抓好金达钼业深加工、东方电解镍及兰凌、喀顺、四合当日产5000吨干法水泥等一批大项目，继续改造和引进一批由初级低选向高级精选、由铁选向冶炼及制造业升级的新项目。

　　在大力发展装备制造业上，争取在运输设备制造业和大型矿山成套设备制造业方面实现率先突破。

　　大力发展现代服务业。完成豪德光彩贸易广场主体工程和配套设施建设，全面展开国内外招商工作；推进新华广场等中心商业街区的建设和改造，吸引更多大型商贸企业落户朝阳；积极发展农村新型流通业态，加快超市、物流配送中转站、综合服务中心建设，把更多的农产品卖出去，把更多的工业品、消费品送下乡。年内全市社会消费品零售总额增长15%以上。

　　巩固创优成果，整合旅游资源，加快市场开发，进一步提升旅游产业地位；充分利用牛河梁红山文化、古生物化石、佛教文化、三燕文化等独特资源，深度包装和实施一批文化产业项目，让历史赋予朝阳独特而珍贵的古文化资源成为经济发展中的特色产业。

　　加快金融服务创新，以社会诚信建设为载体，搞好金融环境建设，防范金融风险；继续深化农村信用社改革，完成城市商业银行挂牌工作；进一步整合担保资源，搞好中小企业信用担保中心扩股工作，积极发展适合农村特点的贷款担保体系。规范保险业公平竞争，加快发展步伐。

　　通过发放廉租住房补贴、改进和规范经济适用房制度、搞活二手房市场等办法，解决困难群众的住房问题，促进房地产市场健康发展。大力整顿和规范市场经济秩序，推进现代市场体系建设。积极拓展现代信息、中介服务、社区服务、教育培训、邮政通信等产业领域。启动建立服务业统计制度，完成农业普查工作。

　　搞好机场复航工作，建设中国民航大学朝阳飞行学院。

　　（三）大力发展县域经济，扎实推进新农村建设

　　叫响县域崛起目标。充分发挥县域经济在新农村建设中的重要载体作用，按照在全省排名中整体位次大幅度前移的要求，进一步提升县域经济在全市经济中的比重。县域生产总值增长25%以上，具备条件的县（市）区实现更快发展；财政一般预算收入增长30%左右；其他主要经济指标增幅达到或超过全省平均水平。

　　充分发挥县域农产品资源和矿产资源优势，加快推进资源转化，努力追求资源利用的最大效益。把培育立县企业和规模以上企业作为县域经济发展的重要内容，推动企业特别是资源加工企业集群化发展。

　　放手、放开、放活县域经济发展，从政策、资金、项目等方面对县域经济给予更大支持，提高县级统筹发展能力。

　　依托现有农业资源，推进优势特色产业基地建设。坚持把发展现代农业作为新农村建设的首要任务，以农业和农村经济结构调整为主线，以农产品加工基地建设为中心，深入推进农业主导产业升级行动；加大对专业乡村建设、村企对接、基地和企业对接的支持力度，建立企业与农户更加紧密的利益联结机制，鼓励发展为加工企业提供原料的农产品生产；重点扶持粮食、畜产品、蔬菜、果品、花卉、薯类、食用菌、中药材八大优质特色农产品生产基地建设，规划和发展酒葡萄种植、可供加工和出口的蔬菜等含税农业，累计建成各类专业乡137个、专业村881个。

　加快畜禽品种改良步伐，加强重大疫病防治，牛羊出栏、育肥规模力求实现更高增长。

　　继续搞好现有保护地棚内种植结构调整，不断提高产出效益；抓好设施农业小区建设，新增保护地面积8万亩。

　认真贯彻落实《农产品质量安全法》，全面提升无公害、绿色、有机农产品的标准化生产水平。

不断改善农村基础设施条件。加大生态文明示范村镇建设力度，在基本实现通油路、通班车、通广播、通电视、通电话、通安全饮用水目标的同时，所有示范村镇都争取基本建成农业主导产业专业乡、专业村。以文明生态村建设为载体，深入搞好以“五清五改一绿”为主要内容的村容镇貌整治工作。加强农村环境整治工作，积极推进农村小康环保行动，加强区域性养殖污染治理示范工程和有机食品种植基地建设，搞好再生资源转化和循环利用示范项目，努力创建环境优美镇和环境优美村。

　　着力改善交通条件，积极推进京四高速公路朝阳段建设，开工建设朝赤高速公路；协助铁路部门和其他投资方力争开工建设朝赤铁路新线，启动建设朝阳南站。

进一步加快供电基础设施建设，努力解决好县域经济快速发展的电力需求问题。

　　用先进物质技术条件装备和改造农业，通过发展农业机械化和信息化、推广先进适用技术、扶持农产品特色品牌等措施，提高农业整体素质和综合效益。

　　坚持不懈地推进山区建设和农田基本建设，搞好高效农业配水配电、河道治理和“四荒”资源开发，完成水土保持综合治理面积40万亩、农村能源“一池三改”生态农业模式示范户1万户。

　　（四）做大做强中心城市，加快城镇化步伐

　　中心城市是区域经济综合实力的集中体现，是区域经济之间竞争的核心平台。实现城乡统筹发展，必须坚持工业反哺农业、城市支持农村的方针，进一步增强中心城市的辐射带动作用和支持调控力，加快推进全市城镇化步伐。

　　坚持扩容提质，做大中心城市。按照“高起点、大手笔”的要求，完成城市空间发展战略及重点发展地区城市设计方案国际竞赛，在城市发展战略上实现“南进”、“北引”，加大面向沿海的基础设施规划建设力度，推进城市总体规划新一轮高水平修编。

　　坚持“面”上求拓展，全面完成北大街改造工程，推进西部工业园、凤凰组团基础设施建设，搞好南塔广场改造，启动建设临空功能区，拉开中心城市框架。

　　突出“线”上抓通畅，启动修建黄河路大桥、木头营子大桥，贯通友谊大街、凌凤路，改造光明街、柳城路等主要街路，完成出口路绿化美化任务，实现城市道路网络对接和交通提档升级。

　　立足“点”上出精品，完成燕都大厦、兴隆商厦等项目主体工程，启动建设盘龙购物广场二期工程，搞好新华广场地下人防工程和辽西博物馆、图书馆、体育馆、古生物化石地质公园建设。

　　发展市区经济，做强中心城市。按照“规划共绘、设施共建、产业共兴、资源共享”的思路，把市区和朝阳县柳城园区作为一个有机整体，统一规划，统一布局，统一指导，统一协调，使市区发展走上功能科学定位、资源合理配置、市场有序竞争、产业协调发展的新路子。

强化产业支撑，提高城市产业竞争力。在抓好市区骨干企业发展的同时，加快园区发展步伐，形成市开发区、高新区、现代农业园区、冶金工业园和双塔、龙城、朝阳县工业园区“七区联动”发展格局；以提升商贸核心区为重点，加快打造中心城区现代商圈；以大力发展新兴工业、都市农业为重点，积极打造卫星城镇带，尽快在市区经济中形成产业特色鲜明、支撑作用明显、三次产业协调发展的新格局。

　　按照适度放权、授权的原则，合理划分市区两级管理权限，适当扩大区级政府在城市建设、管理等方面的自主权，提高区级政府工作的积极性。

　　搞好县城和重点小城镇建设。进一步调整和完善县城和县域城镇体系的规划及修编，启动京四、朝赤两条高速公路节点所在村镇的总体规划编制和重点基础设施项目规划设计工作。坚持以园扩城、以商扩城、以改扩城、以校扩城、以大型企业扩城等多措并举，迅速拉开县城骨架，扩大县城规模。举全市之力支持朝阳县城搬迁，尽快结束朝阳县有县无城的历史。加快北票棚户区改造步伐。采取综合措施，加快重点小城镇建设，努力培育工业强镇、商贸富镇、交通枢纽重镇和旅游休闲大镇。

加强城镇管理。进一步完善城市综合执法运行机制和城镇管理长效机制，推动城管重心向小街、小巷、小区和城乡结合部延伸。积极开展“门前三包”活动月，切实解决好城市脏乱差、违临建等群众反映强烈的问题。推进社区软硬件建设，强化社区物业管理。充分发挥土地储备中心、建投公司的主体作用，增加土地收益，提高全市城镇开发经营水平。加快公用事业的市场化改革，积极盘活城镇公共资源和无形资产。实施二次加压泵站统一管理，着手解决市区二次供水问题。

积极开拓建筑市场，加强工程质量管理，加快发展建筑业。

　　（五）注重民生建设，着力解决好人民群众最关心、最直接、最现实的利益问题

　　打好农村扶贫攻坚战。多渠道筹集资金，加大社会帮扶力度，狠抓户有稳定收入项目建设，大力实施扶贫培训工程，强力推进整村推进和移民搬迁，基本解决现有贫困人口的稳定温饱问题。

千方百计促进就业再就业。在确保有就业能力和就业愿望的零就业家庭至少实现一人稳定就业的基础上，进一步增加零就业家庭就业人员数量，提高稳定就业比重和工资标准。

充分利用现有职教资源，搞好普惠制培训、订单培训、因需施训，新增实名制就业5万人，完成实名制劳务输出33万人次、有组织劳务输出16.5万人次以上。继续为“4050”人员提供公益性岗位，并落实养老、失业、医疗保险补贴政策。

　　进一步完善社会保障体系。继续做好城镇职工养老、失业、医疗、工伤及生育保险的扩面工作，尽快使社会保险覆盖到各种所有制企业。加强社会保险费的征缴和监管，确保各项保险费按时足额发放。发展和谐劳动关系，认真落实农民工和失地农民社会保障工作；搞好农村特困群众生活救助、城乡困难群众医疗救助，研究对农村低保户和城市特困户家庭高中生给予适当救助，做好优抚安置、社会互助和法律援助等工作。

办好人民群众关心的15件实事。一是提高全市企业离退休人员养老金标准，并做到按时足额发放。二是提高全市新型农村合作医疗人均保障标准，以户为单位参合率提高到86%，农村低保人口参合率达到100%。三是提高城市45万低保重点人口保障标准。四是新建九年一贯制寄宿学校10所，完成农村中小学危房改造5万平方米，完成50所农村中小学布局调整。五是全面完成农村贫困户危房修建任务。六是解决偏远农村4万人的安全饮水问题，为200户高氟水农户免费安装除氟器。七是改造农村乡镇卫生院105所。八是完成农村公路网路基改造1000公里，建设黑色路面200公里。九是新建农村“万村千乡市场工程”农家店140家。十是解决国企改制期间并轨遗留的3.5万人就业安置问题。十一是完成“城中村”改造20万平方米。十二是解决全市社区工作人员“两险”待遇，提高工资标准。十三是启动实施第三高中扩建工程。十四是加快客运网络化建设，实现100%行政村通班车。十五是落实大中型水库移民后期扶持政策，确保资金按时、按规定发放到移民手中。

　　（六）坚持经济与社会统筹发展，大力推进和谐社会建设

　　繁荣各项社会事业。围绕农产品、矿产资源深加工，加强产学研联合，组织好科技项目和技术需求对接活动；开展好企业自主创新试点工作，大力实施品牌战略，建设一批科技成果转化基地，培育和引进一批高科技企业，增强科技对经济发展的支撑力。深入实施人才强市战略，搞好政策调整，引进和培养高层次紧缺人才和农村适用型人才。

　　切实把教育摆在优先发展的位置，认真贯彻《义务教育法》，建立完善农村义务教育经费保障机制，进一步巩固和提高“普九”成果；大力发展高中阶段教育，初中升普通高中比例达到50%；加快职业技术教育产业化进程，巩固县级职教中心标准化建设成果，启动朝阳师专和工业学校校址搬迁工作，规划整合一所高水平、现代化的职教学院。

　　加快建立覆盖城乡居民的基本卫生保健制度，以城市社区卫生服务体系和农村预防保健体系建设为重点，进一步完善公共卫生服务体系；加大对医疗机构的监管力度，鼓励发展民办医疗机构，逐步缓解城乡群众“看病难、看病贵”问题。

　　大力发展文化体育事业，加大对公益性文化事业的投入和扶持力度，普及全民健身活动，不断提高竞技体育水平。继续强化文化市场的建设和管理，加强对网吧的治理。积极做好牛河梁红山文化遗址“申遗”工作，办好第二届全民健身节暨首届龙舟大赛。

　　大力支持民族地区经济和社会事业发展，依法管理宗教事务。

　　坚持政府统筹，推进妇女和儿童发展规划的贯彻实施。关心支持残疾人和老龄事业，扎实做好民兵预备役、气象、地震、档案、地方志等各项工作。

实现人口、资源、环境协调发展。进一步完善惠及计划生育家庭的利益导向机制，抓好政策落实，深化出生人口性别比升高问题综合整治，完善“选先评差”机制和责任追究制度，人口出生率控制在9.6‰以内，计划生育率稳定在98%以上。

　　把强化政府责任作为节能降耗、污染减排的关键环节，继续整顿矿业秩序，加大资源勘查和整合力度；依法有效管理古生物化石资源，实行政府主导下的有序开发和保护；大力发展循环经济，推进节能、节水、节地、节材和资源的综合利用；继续加大人工增雨、防雹工作力度，加强云水资源开发利用，强化水资源管理、保护和优化配置；全面启动创建国家环境保护模范城工作，积极开展环保专项整治和“保护母亲河，喝上干净水”活动，继续搞好拆小并大联片供暖，确保市区空气质量二级指标天数全年稳定在85%以上。

　　以治理林业“三乱”为重点，加大森林资源保护力度；坚持封山禁牧，完成造林绿化100万亩；加快推进京四高速公路朝阳段两翼绿化工程，确保高速公路和绿化带建设同步推进；全面规划并适时启动凤凰山、麒麟山迎大凌河面的绿化工作，加快生态朝阳建设步伐。

加强民主法制建设。坚持向人大及其常委会报告和向政协通报重要工作和重大事项制度，认真执行人大通过的各项决议，随时接受人大的工作监督、法律监督和政协的民主监督。认真办理人民代表议案、建议和政协提案，充分听取各民主党派、无党派人士和人民团体的意见建议。深入开展“五五”普法活动，增强公民法制观念和依法维权、依法办事的自觉性。依法管理和规范社会组织、社会事务，协调各种利益关系，促进社会公平正义。进一步加强农村基层政权建设，高标准完成村委会换届工作。

积极推进精神文明建设。深入开展以“加快树立朝阳发展新形象”为主题的思想教育活动和各种群众性精神文明创建活动；认真贯彻落实《公民道德建设实施纲要》，引导群众树立社会主义荣辱观，不断增强全民文明素质，提高城乡文明程度，努力把朝阳建设成为友爱之城。实现赵尚志烈士颅骨回归安葬，筹备建设各种纪念设施。加强国防教育，推进双拥工作。

　　切实维护社会稳定。深入开展社会治安综合治理，继续推进平安创建活动，建立健全社会治安防控体系，依法严厉打击各种违法犯罪活动，坚决扫除“黄赌毒”等社会丑恶现象，提高群众对社会治安的满意率；全面加强外来流动人口及出租屋管理，建设平安和谐社区；严格落实安全生产责任制，及时排除各种安全隐患，坚决遏制重特大安全事故发生；健全社会舆情汇集和分析机制，畅通信访渠道，理顺民气，依法规范上访行为，充分发挥信访联席会议作用，及时妥善处理上访问题及群体性事件。加强突发公共事件应急管理体系建设，提高保障公共安全和处置突发公共事件的能力。

全面提高政府工作效能

　　行政效能是政府体制、工作作风、行政能力和效益的综合反映。加强行政效能建设，是政府履行职责、提高工作效率、全面完成各项工作任务的重要保证。各级政府要按照“为民、务实、清廉”的要求，以解决好执行力弱和软环境差为突破口，全面提高政府工作效能。

提高效能，要体现在开拓创新上。加快发展，需要破除小胜即满、安于现状的思想；奋力赶超，需要抛弃因循守旧、固步自封的陋习。要把创新贯穿于政府工作的始终，努力用新的视角来审视我们的发展思路，用新的思维来分析我们面临的形势，用新的办法来解决我们前进中遇到的各种矛盾和问题。要创新体制机制，深化行政管理体制改革和行政审批制度改革，推进电子政务建设，坚决改变一切束缚发展的做法和规定，促进政府职能转变，创新政府运行机制和管理方式，以与时俱进的精神状态、永续创新的更高要求，开创政府工作新局面。

提高效能，要体现在依法行政上。认真贯彻实施《行政许可法》和《朝阳市全面推进依法行政规划》，严格依法行使权力、履行职责，保证政府行为合法适当。全面推行行政执法责任制、评议考核制和过错责任追究制，坚决纠正、查处违法和不当的行政行为。进一步规范政府重大决策行为，健全科学、民主、依法决策机制，对与群众利益密切相关的重大决策，通过多种渠道，采取多种形式，认真听取人大代表、政协委员的意见，广泛征询社会各界人士的建议，使政府的决策行为更加科学化、民主化、法制化和规范化。进一步完善政务公开制度，扩展政务公开范围，规范政府公开程序，切实保障人民群众知情权、选择权、参与权和监督权。以加强“五型”机关建设为载体，通过学习、培训、管理等途径，全方位提高公务员队伍综合素质，在政府机关营造“人人讲效能、处处抓效能、事事创效能”的良好氛围。

提高效能，要体现在改进作风上。按照“诚信、透明、简化、明确、高效”的要求，不断规范行政行为，进一步提高办事效率和工作水平。诚信，就是树立诚信形象，强化纪律约束，推行服务承诺制，做到一诺千金，凡是承诺的事情，一定确保做到。透明，就是扩大政务公开，实行“阳光操作”，不断提高政府工作透明度。简化，就是善于把复杂问题简单化，简化办事程序，提高办事效率。明确，就是推行各级领导责任制和首问负责制，对群众和企业提出的问题，有明确的态度、明确的答复，决不模棱两可，更不敷衍推诿。高效，就是做各项工作都要雷厉风行，按照“马上办”的要求，以最快的速度“办上马”。

提高效能，要体现在勤政廉政上。坚持求真务实、真抓实干，深入基层，深入群众，真心实意为群众办实事、做好事、解难事，做到群众的利益高于一切，关心群众的疾苦重于一切，解决群众的困难先于一切。牢固树立正确的政绩观，坚决反对官僚主义和形式主义，坚决反对做表面文章，决不搞华而不实、劳民伤财的“形象工程”和“政绩工程”。大力弘扬艰苦奋斗、勤俭节约的优良传统，坚决遏制铺张浪费、奢侈攀比之风。按照“一岗双责”的要求，认真落实党风廉政建设责任制各项规定，建立健全教育、制度、监督并重的惩治和预防腐败体系，努力从源头上预防和治理腐败。深入开展专项治理，重点治理工程招投标、矿产开发、土地出让、产权交易、医药购销和政府采购等领域商业贿赂问题；继续以“双评”活动为载体，大力推进政风行风建设，坚决纠正部门和行业不正之风，以廉政建设的实际成效取信于民。

　　各位代表，朝阳是一方干事创业的热土，是一个有着无限发展潜力和广阔发展前景的城市。实现朝阳跨越式发展，是历史赋予我们的庄严使命，是全市各族人民的殷切期盼。尽管前进的道路不会平坦，但任何困难都动摇不了朝阳人民加快发展的决心！新的一年里，我们将与全市各族人民一道，高高扬起朝阳的旗帜，用心血和汗水开辟一片造福于人民的崭新天地，干出一番无愧于时代的辉煌业绩。我们所做的一切，一定要让朝阳千家万户有更多的笑容、更多的温馨！一定要让朝阳在新一轮发展大潮中树立起崭新的形象！一定要让朝阳变得更富、更美、更强！
